# Which approach is preferred in left hepatocellular carcinoma? Laparoscopic versus open hepatectomy using propensity score matching

**DOI:** 10.1186/s12885-018-4506-3

**Published:** 2018-06-19

**Authors:** Jong Man Kim, Choon Hyuck David Kwon, Heejin Yoo, Kyeung-Sik Kim, Jisoo Lee, Kyunga Kim, Gyu-Seong Choi, Jae-Won Joh

**Affiliations:** 1 Department of Surgery, Samsung Medical Center, Sungkyunkwan University School of Medicine, 81 Irwon-Ro, Gangnam-Gu, Seoul, 06351 Republic of Korea; 2 Biostatistics and Data Center, Samsung Medical Center, Seoul, Republic of Korea

**Keywords:** Hepatocellular carcinoma, Hepatectomy, Laparoscopy, Tumor recurrence, Survival, Minimal invasive surgery

## Abstract

**Background:**

Laparoscopic liver resection has been reported as a safe and effective approach for the management of hepatocellular carcinoma (HCC). However, its perioperative and oncological outcomes have not been evaluated in left hepatectomy patients. The aim of the present study is to compare the outcomes of left hepatectomy through laparoscopic and open approaches in left HCC.

**Methods:**

From December 2012 to October 2016, laparoscopic left hepatectomy (LLH) was performed in 40 patients and open left hepatectomy (OLH) was performed in 80 patients. All clinical data were analyzed retrospectively. Propensity score matching of patients in a 1:1 ratio was conducted based on tumor size and presence of microvascular invasion.

**Results:**

Tumor size and presence of microvascular invasion were higher in the OLH group than the LLH group (*P* < 0.05). However, the operative time was longer in the LLH group than in the OLH group (266 min vs. 239 min; *P* = 0.005). The median postoperative hospital stay was significantly shorter in the LLH group than in the OLH group before and after matching (9 days vs. 13 days; *P* < 0.001). The incidence of complications in the LLH and OLH groups was 10.0 and 7.5%, respectively. The disease-free survival (DFS) and overall survival (OS) in the LLH group were not different from those in the OLH group after propensity score matching.

**Conclusions:**

A laparoscopic approach is feasible and safe for left HCC. The oncologic outcome of LLH is comparable to that of OLH.

## Background

Laparoscopic liver surgery requires additional advanced skills over open surgery. Since the first laparoscopic liver resection in the 1990s, there has been continuous improvement in laparoscopic techniques and devices, and accumulating data have allowed the development of laparoscopic liver resection of hepatocellular carcinoma (HCC) in cirrhotic patients [1–3]. Despite the technical difficulties, the number of publications on laparoscopic liver resection has been increasing [4]. However, many of the studies involve small numbers of patients; thus, evidence supporting its full development is still insufficient. Recent studies have confirmed that laparoscopic HCC resection and laparoscopic donor hepatectomy are safe and seem to improve the postoperative course [5–7].

Significant advantages of laparoscopic wedge resection or left lateral sectionectomy versus open procedures have been widely reported [4, 8, 9]. However, laparoscopic major liver resections have been limited to a few institutions due to the technical demands of the procedure [7, 10–12]. Although a recent study reported that the outcomes of purely laparoscopic right hepatectomy in HCC patients were comparable to those of open right hepatectomy [7], left major hepatectomy has been limited to a few medical centers because of its technical complexity [11]. In addition, left hepatectomy is a less commonly performed surgical procedure compared with right hepatectomy. No report has described the feasibility of pure laparoscopic left hepatectomy in a large cohort of HCC patients.

Previous published studies reported that the oncological outcomes of laparoscopic hepatectomy were comparable to those of open hepatectomy [1, 3, 13]. However, there is insufficient evidence to determine whether laparoscopy is more suitable than an open procedure for the treatment of HCC because of different tumor locations.

In this study, we aimed to compare the outcomes of purely laparoscopic left hepatectomy (LLH) and open left hepatectomy (OLH) in patients with left HCC.

## Methods

### Patients

This study included patients who underwent surgical resection of solitary HCC based on preoperative radiological images between December 2012 and October 2016. This study was approved by the Samsung Medical Center Institutional Review Board (SMC-2017-05-090). A total of 139 patients underwent left hepatectomy because of HCC. The diagnosis of HCC was proved based on pathology after hepatectomy. Ruptured HCC cases (*n* = 5); those with a the history of locoregional therapies such as transarterial embolization (TACE) (*n* = 11), radiofrequency ablation (RFA) (*n* = 2), or the combination of TACE and RFA (*n* = 2); and open conversion cases because of uncontrolled bleeding during laparoscopic procedure (*n* = 1) were excluded. Two clinically comparable groups of patients were studied: those undergoing laparoscopic left hepatectomy (*n* = 40) and those undergoing open left hepatectomy (*n* = 80). The study included hepatectomy from four surgeons. Selection criteria for laparoscopic approach were surgeon dependent. One surgeon did not perform any laparoscopic approach, but three surgeons performed both approaches. Open hepatectomy was performed in cases with previous abdominal surgery or large tumor and in patients who did not agree to undergo LLH because of the expense.

Demographic, preoperative laboratory, and pathologic data were prospectively collected in the electrical medical records. None of the patients received postoperative adjuvant therapy before recurrence. The procedures used for surveillance after liver resection have been described previously [14].

### Laparoscopic left hepatectomy

All liver resections were intended to be totally laparoscopic and were performed according to the described procedures and the surgeon’s usual practice. The patient was placed in a supine position with the legs apart. Pneumoperitoneum was created by carbon dioxide insufflation at a pressure of 11–12 mmHg, and a 0-degree flexible laparoscope camera was used. When the tumor was located in segment 4, an intraoperative sonographic examination was performed to confirm the exact tumor location and its relationship to major blood vessels. Parenchymal transection was performed with the different types of energy devices (Sonicison, Medtronics or Harmonic Ace, Ethicone or Ligasure, Medtronics) in accordance with the surgeon’s usual practice; devices used were advanced bipolar device, and/or cavitron ultrasonic surgical aspirator (CUSA. EXcel, Valleylab, Boulder, CO). The corresponding Glissonean branch was managed using individual vessel ligation or temporary inflow control of the Glisson (TICGL) method according to the surgeon’s preference [15]. A temporary increase of intra-abdominal pressure of up to 15 mmHg was used to balance the central venous pressure in case of hepatic vein bleeding. Small vessels were controlled with bipolar coagulation and larger vessels were clipped or electively stapled. Pedicle clamping was not used routinely, but only when there was bleeding or when a long operation time was anticipated. The specimen was removed through in a small low-midline incision followed vertical extension of umbilical port trochar site or a Pfannenstiel incision unless there was a previous laparotomy scar, in which case the previous incision was used. Drain catheter was routinely placed at left upper quadrant.

### Open left hepatectomy

Open left hepatectomy was performed through a reverse L-incision. After exploration of the abdominal cavity, the anterior approach was applied to dissect and clamp the left Glissonean pedicle below the hilar plate. When the tumor was located in segment 4, intraoperative sonographic examination was performed to confirm the exact tumor location and its relationship to major blood vessels. Parenchymal transection was achieved using a cavitron ultrasound surgical aspirator (CUSA EXcel; Valleylab, Boulder, CO, USA). Individual vessel ligation of hepatic artery and portal vein before parenchymal transection was performed and intermittent inflow control was done when necessary. Hemostasis was achieved by monopolar electrocoagulation, argon beam, clips, or non-absorbable sutures. Systematic routine placement of an abdominal drain was performed during surgery.

### Pathology

Postoperative histological assessment included maximal tumor size, encapsulation, intrahepatic metastasis, multicentric occurrence, microvascular invasion, serosal involvement, and cirrhosis. The histologic grade of HCC was assigned according to the Edmonson-Steiner system as well differentiated (grade I), moderately differentiated (grade II), or poorly differentiated (grade III, IV).

### Statistical analysis

All statistical analyses were performed using SAS version 9.4 (SAS Institute Inc., Cary, NC, USA). Continuous variables are described as median with range. Categorical variables are expressed as number and percentage of patients. Fisher’s exact test was conducted to evaluate differences in the frequencies of categorical variables between the groups. Mann-Whitney *U* analysis was conducted to evaluate differences in continuous variables between the two groups. The Kaplan-Meier survival method was performed to evaluate differences in patient survival between the two groups. Prognostic factors of patient survival were identified by Cox regression analysis. To overcome possible selection bias, 1:1 propensity score matching between the laparoscopic left hepatectomy and open left hepatectomy cohorts was applied using multiple logistic regression and a 1:1 matching requirement via the nearest-neighbor matching method. Statistical matching was executed using R 3.2.1 (Vienna, Austria; http://www.r-prject.org/). We matched patients with regard to tumor size and presence of microvascular invasion. All tests were two-sided, and statistical significance was defined as *P* < 0.05.

## Results

### Baseline characteristics

The LLH group contained 40 patients, and the OLH group contained 80 patients. All patients underwent curative hepatectomy. Patient baseline and preoperative characteristics of the two groups are summarized in Table 1. Gender, age, etiology, white blood cell count, neutrophil-lymphocyte ratio, hemoglobin level, platelet count, total bilirubin, alkaline phosphatase (ALP), international normalized ratio (INR), albumin, creatinine, C-reactive protein (CRP), alpha-fetoprotein (AFP), and indocyanine green retention rate at 15 min (ICG-R15) were not different between the two groups before and after matching. The median aspartate transaminase (AST) and protein induced by vitamin K absence/antagonism-II (PIVKA-II) were higher in the OLH group than in the LLH group (31 U/L vs. 28 U/L; *P* = 0.041 and 278.5 mAU/mL vs. 32.5 mAU/mL; *P* = 0.001), but there were no statistically significant differences between the two groups after matching. There was no difference in alanine transaminase (ALT) level between the two groups before matching, but ALT was higher in the OLH group than in the LLH group after matching.

**Table 1:** Baseline characteristics

	Before matching			After matching		
	OLH (*n* = 80)	LLH (*n* = 40)	*P*-value	Open (*n* = 37)	Laparoscopic (*n* = 37)	*P*-value
Gender (male)	68 (85.0%)	31 (77.5%)	0.319	31 (83.8%)	30 (81.1%)	0.705
Age	58 (29–80)	59 (34–78)	0.269	55 (29–79)	58 (34–78)	0.203
Etiology			0.253			0.321
NBNC	17 (21.3%)	7 (17.5%)		3 (8.1%)	6 (16.2%)	
HBV	58 (72.5%)	29 (72.5%)		31 (83.8%)	27 (73.0%)	
HCV	2 (2.5%)	4 (10.0%)		0 (0%)	4 (10.8%)	
Alcohol	3 (3.8%)	0 (0%)		3 (8.1%)	0 (0%)	
WBC (/μL)	5780 (2070–10,840)	5370 (3660–8870)	0.334	5240 (2070–9690)	5480 (3660–8870)	0.912
NLR	0.61 (0.23–1.53)	0.69 (0.17–1.30)	0.054	0.67 (0.26–1.53)	0.72 (0.17–1.30)	0.294
Hemoglobin (g/dL)	14.2 (8.4–17.0)	14.2 (9.6–17.2)	0.743	14.1 (8.4–17.0)	14.3 (9.6–17.2)	0.947
Platelets (/μL)	172,500 (44,000–397,000)	177,500 (83,000–302,000)	0.892	168,000 (44,000–266,000)	180,000 (83,000–259,000)	0.662
Total bilirubin (mg/dL)	0.6 (0.2–1.8)	0.5 (0.2–1.5)	0.993	0.6 (0.2–1.8)	0.5 (0.2–1.5)	0.341
AST (U/L)	31 (14–120)	28 (16–80)	0.041	32 (14–120)	25 (16–69)	0.055
ALT (U/L)	27 (5–254)	24 (11–100)	0.467	34 (5–254)	24 (11–100)	0.018
ALP (U/L)	74 (38–155)	65 (40–177)	0.065	76 (48–132)	64 (41–177)	0.063
INR	1.04 (0.87–1.27)	1.03 (0.87–1.60)	0.495	1.04 (0.96–1.27)	1.03 (0.87–1.60)	0.618
Albumin (g/dL)	4.4 (3.2–5.2)	4.5 (4.0–5.2)	0.196	4.3 (3.4–5.2)	4.5 (4.0–5.2)	0.098
Creatinine (mg/dL)	0.89 (0.50–2.08)	0.91 (0.51–4.21)	0.330	0.88 (0.56–2.08)	0.91 (0.51–4.21)	0.319
CRP (mg/dL)	0.09 (0.03–3.68)	0.07 (0.03–0.42)	0.427	0.11 (0.03–3.68)	0.07 (0.03–0.42)	0.250
AFP (mg/dL)	33.8 (1.3–200,000)	11.8 (1.3–19,481)	0.124	13.8 (1.3–14,841)	13.0 (1.3–19,481)	0.541
PIVKA-II (mAU/mL)	278.5 (12–75,000)	32.5 (13–3695)	0.001	43 (12–1270)	33 (15–2685)	0.569
ICG-R15 (%)	9.8 (4.2–20.7)	9.3 (2.1–37.1)	0.689	8.0 (4.2–18.2)	9.3 (5.1–37.1)	0.173

*OLH open left hepatectomy, *LLH* laparoscopic left hepatectomy, *NBNC* non B non C, *HBV* hepatitis B virus, *HCV* hepatitis C virus, *WBC* white blood cells, *NLR* neutrophil-lymphocyte ratio, *AST* aspartate transaminase, *ALT* alanine transaminase, *ALP* alkaline phosphatase, *CRP* C-reactive protein, *AFP* alpha-fetoprotein, *PIVKA-II* protein induced by vitamin K absence/antagonism-II, *ICG-R15* indocyanine green retention rate at 15 min

### Perioperative and pathologic characteristics

The median operation time in the LLH group was longer than that in the OLH group (266 min vs. 239 min; *P* = 0.005), but no statistically significant difference was found between the two groups after matching (Table 2). Blood loss was not different between the two groups before and after matching. Two patients (2.5%) in the OLH group and three patients (7.5%) in the LLH group received red blood cells before matching. The median postoperative hospital stay was significantly shorter in the LLH group than in the OLH group before and after matching (9 days vs. 13 days; *P* < 0.001).

**Table 2:** Perioperative and pathologic characteristics

	Before matching			After matching		
	Open (*n* = 80)	Laparoscopic (*n* = 40)	*P*-value	Open (*n* = 37)	Laparoscopic (*n* = 37)	*P*-value
Perioperative						
Operative time (min)	239 (99–599)	267 (141–509)	0.005	239 (99–599)	267 (141–509)	0.129
Blood loss (mL)	300 (100–1700)	275 (50–2000)	0.230	300 (100–1700)	250 (50–2000)	0.468
RBC transfusion	2 (2.5%)	3 (7.5%)	0.332	2 (5.4%)	2 (5.4%)	0.337
RBC transfusion (unit)	2.5 (1–4)	2 (1–2)	0.201	2.5 (1–4)	1.5 (1–2)	0.375
Hospitalization (days)	13 (6–71)	9 (5–21)	< 0.001	13 (6–45)	9 (5–21)	< 0.001
Pathologic						
Tumor size (cm)	4.2 (0.9–14)	2.6 (0.6–11.5)	< 0.001	2.8 (1.1–10)	2.8 (0.9–11.5)	0.225
Free resection margin (mm)	10 (1–60)	15 (1–65)	0.173	13 (1–50)	15 (1–65)	0.476
Tumor necrosis	38 (47.5%)	13 (32.5%)	0.170	15 (40.5%)	12 (32.4%)	0.466
Tumor hemorrhage	46 (57.5%)	18 (45.0%)	0.245	17 (46.0%)	16 (43.2%)	0.808
Encapsulation			0.598			0.292
None	5 (6.3%)	4 (10.3%)		2 (5.4%)	4 (11.1%)	
Partial	20 (25.3%)	11 (28.2%)		9 (24.3%)	10 (27.8%)	
Complete	54 (68.4%)	24 (61.5%)		26 (70.3%)	22 (61.1%)	
Microvascular invasion	64 (81.0%)	24 (61.5%)	0.027	25 (67.6%)	23 (63.9%)	0.730
PVTT	12 (15.2%)	5 (12.8%)	1.000	3 (8.1%)	4 (11.1%)	0.680
BDTT	4 (5.1%)	0 (0%)	0.301	3 (8.1%)	0 (0%)	1.000
Serosal involvement	2 (2.5%)	0 (0%)	1.000	0 (0%)	0 (0%)	1.000
Intrahepatic metastasis	9 (11.4%)	3 (7.7%)	0.749	4 (10.8%)	2 (5.6%)	0.440
Multicentric occurrence	4 (5.1%)	1 (2.6%)	1.000	2 (5.4%)	1 (2.8%)	0.586
Cirrhosis	33 (41.8%)	18 (46.2%)	0.696	20 (54.1%)	15 (41.7%)	0.260

*OLH open left hepatectomy, *LLH* laparoscopic left hepatectomy, *RBC* red blood cells, *PVTT* portal vein tumor thrombosis, *BDTT* bile duct tumor thrombosis

The overall complication rate was 10.0% (*n* = 8) in the OLH group and 7.5% (*n* = 3) in the LLH group (*P* = 0.468). Atrial fibrillation (*n* = 1), ascites (*n* = 1), increased total bilirubin level (*n* = 1), nausea (*n* = 2), pleural effusion (*n* = 1), and pulmonary artery embolization (*n* = 1) developed in the OLH group and ascites (*n* = 1), cardiac enzyme elevation (*n* = 1), and pleural effusion (*n* = 1) in the LLH group. However, none of the patients had complications greater than Clavien–Dindo classification III. All complications were controlled with pharmacologic treatment or conservative management.

Median tumor size in the OLH group was larger than that in the LLH group (4.2 cm vs. 2.6 cm; *P* < 0.001). The incidence of microvascular invasion was higher in the OLH group was higher than in the LLH group (81.0% vs. 61.5%; *P* = 0.027). However, tumor size and microvascular invasion were not different between the two groups after matching. Free resection margin, tumor necrosis, tumor hemorrhage, encapsulation, portal vein tumor thrombosis (PVTT), bile duct tumor thrombosis (BDTT), serosal involvement, intrahepatic metastasis, multicentric occurrence, and cirrhosis were not different between the two groups before and after matching (Table 2).

### Tumor recurrence and survival

**Fig. 1:**
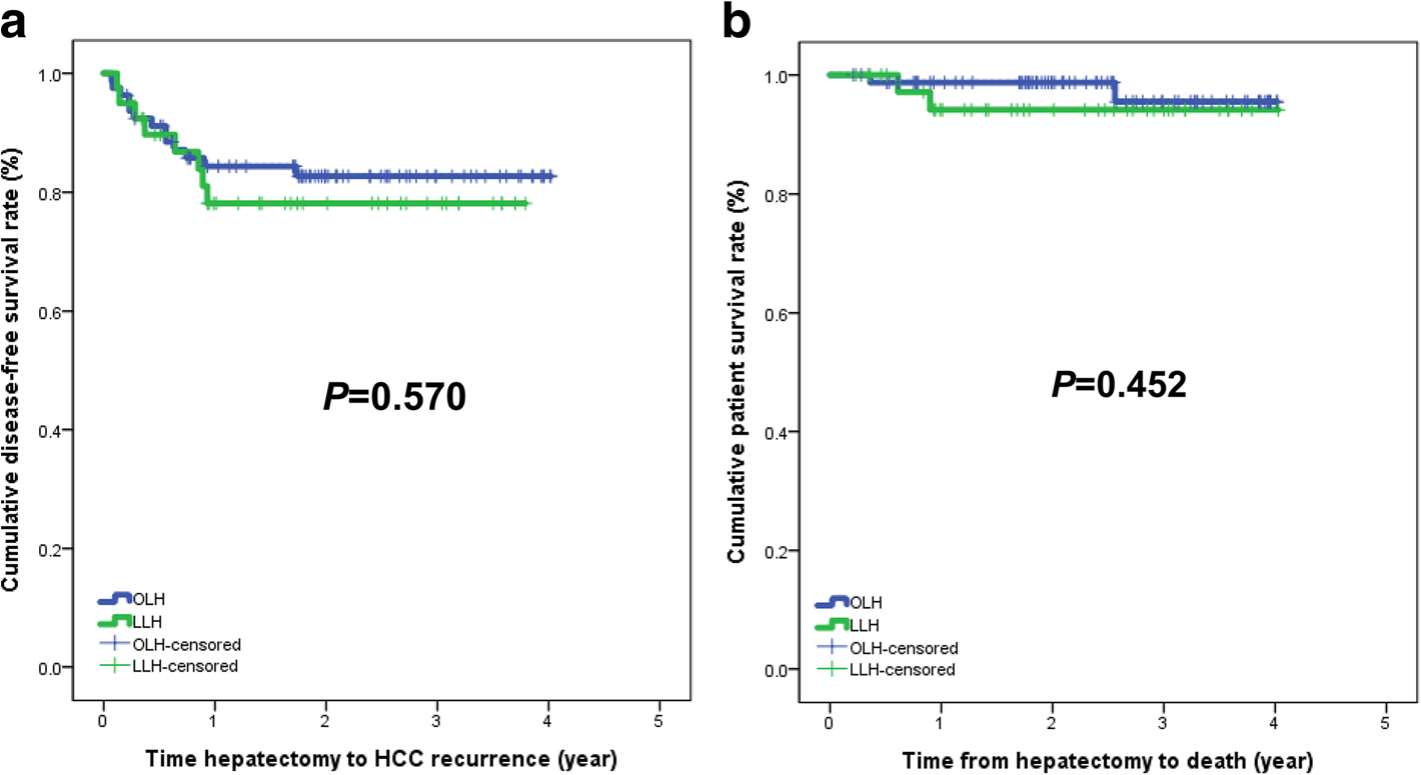
**a** Disease-free survival and **b** patient survival before propensity score matching

**Fig. 2:**
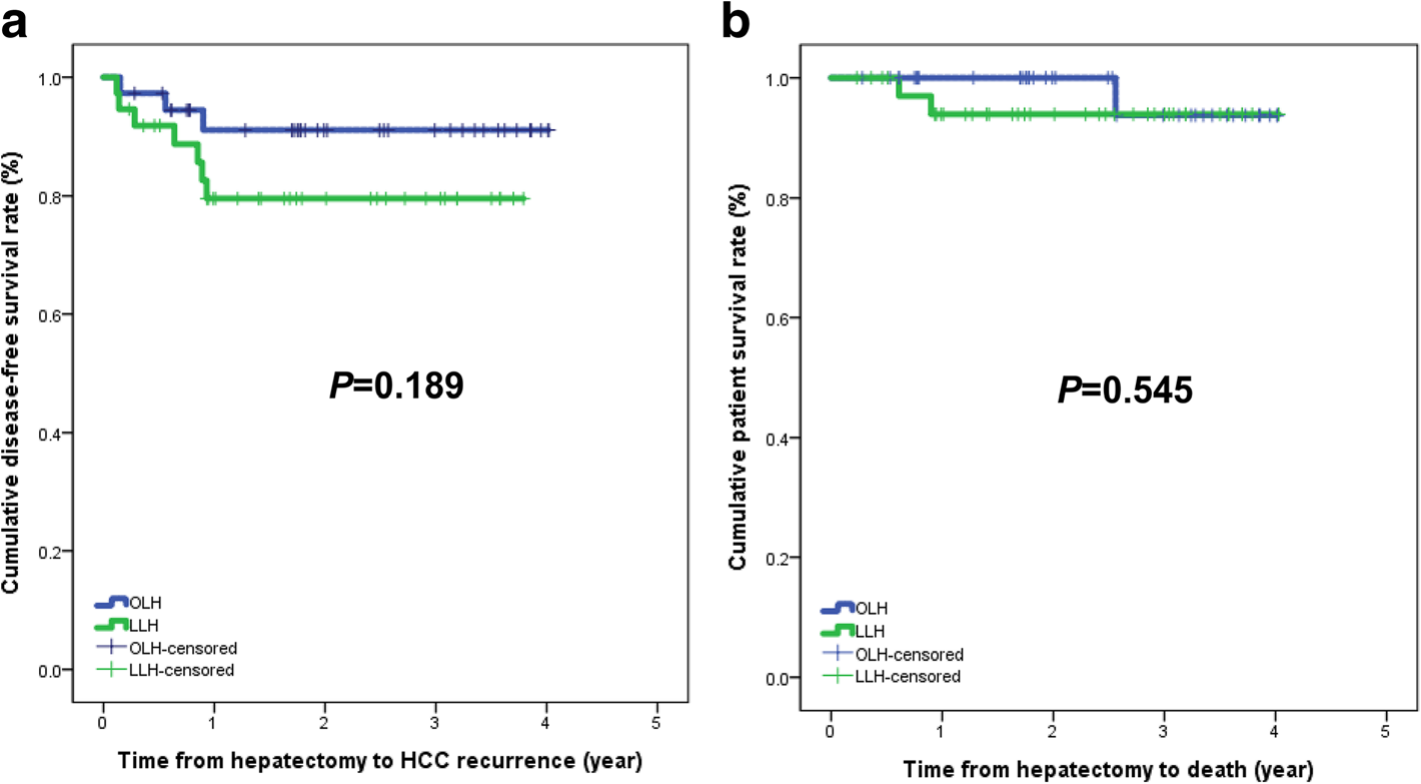
**a** Disease-free survival and **b** patient survival after propensity score matching

The median follow-up period was 26.0 months (range, 2.5–48.2 months) for the OLH group and 22.8 months (range, 2.8–48.4 months) for the LLH group before matching (*P* = 0.226). Recurrence of HCC was observed in 13 patients (16.3%) in the OLH group and 8 patients (20.0%) in the LLH group. Initial recurrence sites were liver (*n* = 12) and synchronous liver and lung (*n* = 1) in the OLH group. The initial recurrent site in the LLH group was liver in seven patients and peritoneum in one patient. However, no trocar-site deposits were observed in the LLH group. Two patients (2.5%) in the OLH group and two patients (5.0%) in the LLH group died of HCC recurrence. The disease-free survival (DFS) and patient survival (PS) in the LLH group were similar to those in the OLH group before matching (*P* = 0.570 and *P* = 0.452, respectively, Fig. 1). The DFS and PS at 3 years were 79.6 and 93.9% in the LLH group and 91.1 and 93.8% in the OLH group, respectively. The DFS in the LLH group was worse than that in the OLH group after matching, but there was no statistically significant difference between the two groups (*P* = 0.189). The PS in the LLH group was similar to that in the OLH group (*P* = 0.545; Fig. 2).

No risk factors for predicting HCC recurrence were revealed after propensity score matching (Table 3). Laparoscopic approach was not a risk factor of HCC recurrence in univariate and multivariate analysis.

**Table 3:** Risk factors for HCC recurrence in left hepatectomy patients after propensity matching in the univariate analysis

	Hazard ratio	95% CI	*P*-value
Laparoscopic left hepatectomy	2.404	0.683–8.460	0.172
Gender (female)	0.546	0.077–3.885	0.546
Age	0.998	0.958–1.041	0.940
NLR	2.310	0.105–51.005	0.596
Hemoglobin	0.979	0.777–1.235	0.860
Platelets	0.996	0.986–1.006	0.387
AST	1.232	0.610–2.491	0.561
ALT	1.082	0.610–1.920	0.788
ALP	1.715	0.211–13.911	0.614
Albumin	0.358	0.085–1.508	0.161
CRP	0.996	0.690–1.437	0.983
AFP	0.954	0.810–1.123	0.570
PIVKA-II	1.309	0.948–1.806	0.102
ICG-R15	3.038	1.032–8.938	0.044
Tumor size	1.636	0.480–5.579	0.431
Tumor necrosis	1.273	0.351–4.612	0.713
Tumor hemorrhage	0.281	0.060–1.327	0.109
Encapsulation	0.357	0.080–1.590	0.177
Microvascular invasion	1.392	0.423–4.583	0.587
PVTT	3.075	0.538–17.575	0.207
Intrahepatic metastasis	1.646	0.203–13.334	0.641
Multicentric occurrence	2.874	0.496–16.650	0.239
Free resection margin	1.669	0.670–4.160	0.271
Cirrhosis	1.043	0.994–1.008	0.766
Operative time	1.043	0.352–3.088	0.940
RBC transfusion	2.300	0.387–13.673	0.360

*NLR neutrophil-lymphocyte ratio, *AST* aspartate transaminase, *ALT* alanine transaminase, *ALP* alkaline phosphatase, *CRP* C-reactive protein, *AFP* alpha-fetoprotein, *PIVKA-II* protein induced by vitamin K absence/antagonism-II, *ICG-R15* indocyanine green retention rate at 15 min, *PVTT* portal vein tumor thrombosis, *RBC* red blood cells

## Discussion

Laparoscopic liver resection has become more frequent, and the results of large series have been reported worldwide, confirming the technical feasibility, postoperative benefit, and oncological safety of this technique [4, 5, 7, 13]. A recent study reported that laparoscopic liver resection is safe and feasible in patients with solitary large HCC (diameter 5-10 cm) [16, 17]. Nevertheless, the application of the laparoscopic technique to liver resection for HCC is challenging.

Tumor size and location are two important factors determining the indications for laparoscopic liver resection in patients with HCC. We have used the Glissonean approach of left hepatic artery and left portal vein for inflow control in patients with hepatocellular carcinoma [15]. In this study, we focused on only left hepatectomy in patients with solitary HCC because surgical approaches are different depending on tumor location. In addition, the lack of an adequate resection margin can be a problem during non-anatomical hepatectomy when the comparison between laparoscopic and open hepatectomy includes all procedures of hepatectomy.

Our study included solitary HCC patients who were diagnosed in the preoperative radiologic images. In our study, intrahepatic metastasis in 12 patients (10%) and multicentric occurrence in 5 patients (4.2%) were reported in the pathology. There was a slight difference between preoperative imaging and pathologic report. Intrahepatic metastasis or multicentric occurrence were not detected in the preoperative images because of small size.

The present study found that the duration of operation, blood loss, transfusion rate, and operative complication rates were not significantly different between the laparoscopic and open hepatectomy groups after matching. However, the hospitalization stay was shorter in the LLH group than in the OLH group. The open conversion rate in the patients who underwent laparoscopic hepatectomy was 2.3–4.1% in published studies [18, 19]. The conversion rate for LLH in the present study was 2.4% (*n* = 1).

Previous studies are summarized in Table 4. These studies, which included all hepatectomy procedures, reported that blood loss in laparoscopic hepatectomy was lower than that in open hepatectomy [1, 3, 8]. However, another study reported that only left hepatectomy also showed greater blood loss with the laparoscopic approach compared with the open approach [20]. Adequate pressure in the pneumoperitoneum is advantageous for the reduction of venous bleeding during hepatectomy. However, previous studies revealed that blood loss in the laparoscopic approach was higher than in the open approach if pneumoperitoneum was not made in the operative field. These results might reflect the additional difficulties and complexity of laparoscopic hepatectomy. Most previous studies reported similar transfusion rates between the two groups [1–3, 7, 12, 13, 17, 20]. Our study also showed that transfusion rate was similar in both groups.

**Table 4:** Review of published literature on HCC patients

Authors	Study	Group	Operation	Blood loss (mL)	Transfusion (n)	Operative time (min)	Hospital stay (days)	Morbidity (≥Grade III)
Aldrighetti et al. [2]	Retrospective	LR (*n* = 16)	All^a^	258	4	150	6.3	4 (25%) / 1
		OR (*n* = 16)		617 (*P* = 0.008)	6 (*P*=NS)	240 (*P* = 0.044)	9.0 (*P* = 0.039)	7 (43.7%) / 0 (*P*=NS)
Tranchart et al. [1]	Retrospective	LR (*n* = 42)	All^a^	364	4 (9.5%)	233	6.7	10 / 4
	Matching	OR (*n* = 42)		723 (*P* < 0.001)	7 (16.7%) (*P* = 0.51)	221 (*P* = 0.90)	9.6 (*P* < 0.001)	18 / 5
Cheung et al. [3]	Retrospective	LR (*n* = 32)	All^a^	150	0	232.5	4	12 (18.8%) / 12
	Matching	OR (*n* = 64)		300 (*P* = 0.001)	3 (*P* = 0.534)	204.5 (*P* = 0.938)	7 (*P* < 0.001)	2 (6.3%) / 1
Komatsu et al. [13]	Retrospective	LR (*n* = 38)	Right/Left Hepatectomy	100	2	365	7.5	12 (31.6%) / 5
	Matching	OR (*n* = 38)		80 (*P* = 0.094)	1 (*P* = 0.556)	300 (*P* < 0.001)	10.0 (*P* = 0.079)	23 (60.5%) / 7 (*P* = 0.011)
Zhang et al. [20]	Retrospective	LR (*n* = 20)	Left hepatectomy	180	0	143	7	0 (0%)
		OR (*n* = 25)		350 (*P* < 0.05)	0	137 (*P* > 0.05)	12 (*P* < 0.05)	10 (40%) / 2 (*P* < 0.05)
Xiang et al. [17]	Prospective	LR (*n* = 128)	All^a^	456	23 (18.0%)	234	11.4	26 (20.3%) / 12
	Tumor size: 5–10 cm	OR (*n* = 207)		481 (*P* = 0.589)	42 (20.3%) (*P* = 0.602)	236 (*P* = 0.886)	15.8 (*P* < 0.001)	74 (35.7%) / 37 (*P* = 0.003)
Yoon et al. [7]	Retrospective	LR (*n* = 33)	Right hepatectomy	126	0	297	10.0	1
	Matching	OR (*n* = 33)		132 (*P* = 0.613)	0	176 (*P* < 0.001)	13.9 (*P* < 0.001)	7 (*P* = 0.054)

All^a^: All hepatectomy included in the study

Two studies that included only left hepatectomy reported that operative time for the laparoscopic approach was significantly longer than that for open hepatectomy [20]. Three matching studies reported similar operative times between the two groups [1, 3, 17], but two studies showed that operative times were significantly longer in the laparoscopic approach than in the open approach despite matching [7, 12]. All previous studies reported a shorter hospital stay with the laparoscopic approach than the open approach [1–3, 7, 13, 17, 20].

The mortality and morbidity rates of patients who underwent laparoscopic left hepatectomy were 0 and 7.5%, respectively. Our results are better than those of several other reports [1–3, 13]. The present study did not reveal a significant difference in complication rate between the groups because the rate of complications in both groups was very small. Although the difference was not statistically significant, the rate of postoperative complications tended to be higher in the open left hepatectomy group (10.0%) compared with that in the laparoscopic left hepatectomy group (7.5%) (*P* = 0.468). Bile leakage did not develop in any of our cases. With meticulous dissection, good surgical outcomes could be expected. Even after matching, we found that the complications rates of laparoscopic left hepatectomy were comparable to those of open left hepatectomy.

Despite an exponential growth in cases of laparoscopic liver resection, the outcomes in HCC patients are yet to be fully elucidated. To overcome selection bias as much as possible, propensity score matching was employed in this study. The propensity score model reduces the different distribution of covariates among individuals allocated to specific intervention [21]. Although a randomized controlled trial can provide the most unbiased evidence for clinical science, it is unfeasible to recruit patients and obtain consent when the patients have to choose between surgical procedures with obvious differences. A propensity score model is closest to the actual clinical situation and decreases the variance of an estimated exposure effect without increasing the bias.

In the present study, tumor size was larger and presence of microvascular invasion was higher in the OLH group that in the LLH group before matching. Large tumor size as a contraindication to laparoscopic hepatectomy remains controversial. Therefore, we performed propensity score matching using these two variables to compare the oncological outcomes between LLH and OLH.

We showed that DFS was lower in the LLH than in the OLH group after matching, but there were no statistically significant differences in DFS and PS between LLH and OLH. Previous studies reported that DFS and PS in laparoscopic approaches were comparable to those in open approaches (Table 5) [1, 3, 7, 13, 17]. Our study also revealed the similar outcomes between LLH and OLH.

**Table 5:** Survival of HCC patients after laparoscopic or open resection in published studies

Authors	Disease-free survival	Patient survival
Tranchart et al. [1]	1-yr, 3-yr, 5-yr in LR: 81.6, 60.9, 45.6%	1-yr, 3-yr, 5-yr in LR: 93.1, 74.4, 59.5%
	1-yr, 3-yr, 5-yr in OR: 70.2, 54.3, 37.2% (*P* = 0.29)	1-yr, 3-yr, 5-yr in OR: 81.8, 73.0, 47.4% (*P* = 0.25)
Cheung et al. [3]	–	1-yr, 3-yr, 5-yr in LR: 96.6, 87.5, 76.6%
		1-yr, 3-yr, 5-yr in OR: 95.2, 72.9, 57.0% (*P* = 0.142)
Komatsu et al. [13]	3-yr in LR:29.7%	3-yr in LR:73.4
	3-yr in OR: 50.3% (*P* = 0.219)	3-yr in OR: 69.2% (*P* = 0.951)
Xiang et al. [17]	1-yr, 3-yr in LR: 89.4, 67.3%	1-yr, 3-yr in LR: 94.4, 81.4%
	1-yr, 3-yr in OR: 88.7, 66.7% (*P* = 0.902)	1-yr, 3-yr in OR: 93.6, 82.2% (*P* = 0.802)
Yoon et al. [7]	2-yr in LR:85.1%	2-yr in LR:100%
	2-yr in OR:83.9% (*P* = 0.645)	2-yr in OR: 88.8% (*P* = 0.090)

* yr year, *LR* laparoscopic resection, *OR* open resection, DFS

The present study has limitations that include the relatively small sample size, short follow-up duration, and retrospective design. However, our study has the strength of including only left hepatectomy in HCC patients, thus excluding the selection bias of various surgical hepatectomy procedures.

## Conclusions

Present study confirmed the recognized advantage of LLH regarding reduced hospitalization and showed a similar complication rate to OLH. Although the LLH group appears to have a lower DFS than the OLH group, there is no statistical difference in the oncological outcome between the two groups. The present study reveals that pure LLH is safe and feasible in selected patients with solitary and small HCC.

## Abbreviations

AFP: Alpha-fetoprotein
ALP: Alkaline phosphatase
ALT: Alanine transaminase
AST: Aspartate transaminase
BDTT: Bile duct tumor thrombosis
CRP: C-reactive protein
DFS: Disease-free survival
HCC: Hepatocellular carcinoma
ICG-R15: Indocyanine green retention rate at 15 min
INR: International normalized ratio
LLH: Laparoscopic left hepatectomy
OLH: Open left hepatectomy
PIVKA-II: Protein induced by vitamin K absence/antagonism-II
PS: Patient survival
PVTT: Portal vein tumor thrombosis
RFA: Radiofrequency ablation
TACE: Transarterial chemoembolization

## References

[1] Tranchart H, Di Giuro G, Lainas P, Roudie J, Agostini H, Franco D (2010). Laparoscopic resection for hepatocellular carcinoma: a matched-pair comparative study. Surg Endosc.

[2] Aldrighetti L, Guzzetti E, Pulitano C, Cipriani F, Catena M, Paganelli M (2010). Case-matched analysis of totally laparoscopic versus open liver resection for HCC: short and middle term results. J Surg Oncol.

[3] Cheung TT, Poon RT, Yuen WK, Chok KS, Jenkins CR, Chan SC (2013). Long-term survival analysis of pure laparoscopic versus open hepatectomy for hepatocellular carcinoma in patients with cirrhosis: a single-center experience. Ann Surg.

[4] Ciria R, Cherqui D, Geller DA, Briceno J, Wakabayashi G (2016). Comparative short-term benefits of laparoscopic liver resection: 9000 cases and climbing. Ann Surg.

[5] Cheung TT, Dai WC, Tsang SH, Chan AC, Chok KS, Chan SC (2016). Pure laparoscopic hepatectomy versus open hepatectomy for hepatocellular carcinoma in 110 patients with liver cirrhosis: a propensity analysis at a single center. Ann Surg.

[6] Kim KH, Kang SH, Jung DH, Yoon YI, Kim WJ, Shin MH (2017). Initial outcomes of pure laparoscopic living donor right hepatectomy in an experienced adult living donor liver transplant center. Transplantation.

[7] Yoon YI, Kim KH, Kang SH, Kim WJ, Shin MH, Lee SK (2017). Pure laparoscopic versus open right hepatectomy for hepatocellular carcinoma in patients with cirrhosis: a propensity score matched analysis. Ann Surg.

[8] Aldrighetti L, Pulitano C, Catena M, Arru M, Guzzetti E, Casati M (2008). A prospective evaluation of laparoscopic versus open left lateral hepatic sectionectomy. J Gastrointest Surg.

[9] Truant S, Bouras AF, Hebbar M, Boleslawski E, Fromont G, Dharancy S (2011). Laparoscopic resection vs. open liver resection for peripheral hepatocellular carcinoma in patients with chronic liver disease: a case-matched study. Surg Endosc.

[10] Dagher I, O'Rourke N, Geller DA, Cherqui D, Belli G, Gamblin TC (2009). Laparoscopic major hepatectomy: an evolution in standard of care. Ann Surg.

[11] Kluger MD, Vigano L, Barroso R, Cherqui D (2013). The learning curve in laparoscopic major liver resection. J Hepatobiliary Pancreat Sci.

[12] Komatsu S, Brustia R, Goumard C, Sepulveda A, Perdigao F, Soubrane O (2017). Clinical impact of laparoscopic hepatectomy: technical and oncological viewpoints. Surg Endosc.

[13] Komatsu S, Brustia R, Goumard C, Perdigao F, Soubrane O, Scatton O (2016). Laparoscopic versus open major hepatectomy for hepatocellular carcinoma: a matched pair analysis. Surg Endosc.

[14] Kim JM, Kwon CH, Joh JW, Park JB, Ko JS, Lee JH (2013). The effect of alkaline phosphatase and intrahepatic metastases in large hepatocellular carcinoma. World J Surg Oncol.

[15] Lee N, Cho CW, Kim JM, Choi GS, Kwon CHD, Joh JW (2017). Application of temporary inflow control of the Glissonean pedicle method provides a safe and easy technique for totally laparoscopic hemihepatectomy by Glissonean approach. Ann Surg Trear Res.

[16] Gil E, Kwon CHD, Kim JM, Choi GS, Heo JS, Cho W (2017). Laparoscopic liver resection of hepatocellular carcinoma with a tumor size larger than 5 cm: review of 45 cases in a tertiary institution. J Laparoendosc Adv Surg Tech A.

[17] Xiang L, Li J, Chen J, Wang X, Guo P, Fan Y (2016). Prospective cohort study of laparoscopic and open hepatectomy for hepatocellular carcinoma. Br J Surg.

[18] Nguyen KT, Gamblin TC, Geller DA (2009). World review of laparoscopic liver resection-2,804 patients. Ann Surg.

[19] Long TC, Bac NH, Thuan ND, le Dat T, Viet DQ, le CH C (2014). Laparoscopic liver resection: 5-year experience at a single center. Surg Endosc.

[20] Zhang Y, Huang J, Chen XM, Sun DL (2016). A Comparison of laparoscopic versus open left Hemihepatectomy for hepatocellular carcinoma. Surg Laparosc Endosc Percutan Tech.

[21] Austin PC (2011). An introduction to propensity score methods for reducing the effects of confounding in observational studies. Multivar Behav Res.

